# Verification of a National Emission Inventory and Influence of On-road Vehicle Manufacturer-Level Emissions

**DOI:** 10.1021/acs.est.0c08363

**Published:** 2021-03-19

**Authors:** Jack Davison, Rebecca A. Rose, Naomi J. Farren, Rebecca L. Wagner, Tim P. Murrells, David C. Carslaw

**Affiliations:** † Wolfson Atmospheric Chemistry Laboratories, University of York, York YO10 5DD, United Kingdom; ‡ Ricardo Energy & Environment, Harwell, Oxfordshire OX11 0QR, United Kingdom

## Abstract

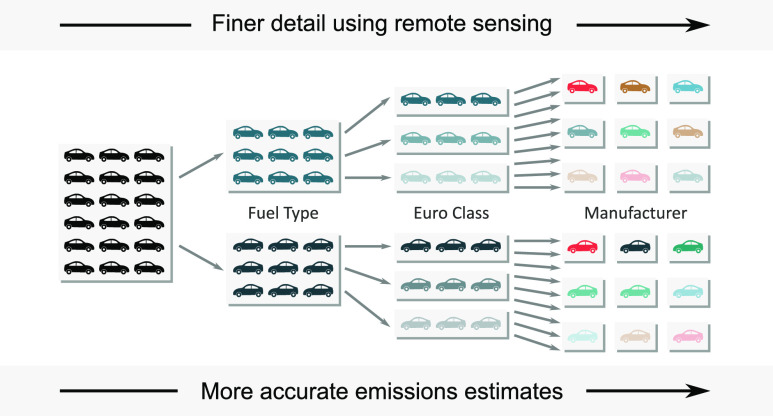

Road vehicles make important contributions to a wide range of pollutant emissions from the street level to global scales. The quantification of emissions from road vehicles is, however, highly challenging given the number of individual sources involved and the myriad factors that influence emissions such as fuel type, emission standard, and driving behavior. In this work, we use highly detailed and comprehensive vehicle emission remote sensing measurements made under real driving conditions to develop new bottom-up inventories that can be compared to official national inventory totals. We find that the total UK passenger car and light-duty van emissions of nitrogen oxides (NO_*x*_) are underestimated by 24–32%, and up to 47% in urban areas, compared with the UK national inventory, despite agreement within 1.5% for total fuel used. Emissions of NO_*x*_ at a country level are also shown to vary considerably depending on the mix of vehicle manufacturers in the fleet. Adopting the on-road mix of vehicle manufacturers for six European countries results in up to a 13.4% range in total emissions of NO_*x*_. Accounting for the manufacturer-specific fleets at a country level could have a significant impact on emission estimates of NO_*x*_ and other pollutants across the European countries, which are not currently reflected in emission inventories.

## 1 Introduction

Emission inventories are an important component of the management of air pollution and provide essential input to air quality models. Emission inventories are required and used at a range of scales from single sources and road sections through to quantifying national total emissions. At the local scale, estimating the emissions along individual road links is required to understand near-road exposures to air pollution. Equally, at a national scale, establishing total emissions is required to meet international obligations, such as the European National Emission Ceiling Directive (NECD).^1^ The accuracy of emission inventories is of central importance for many issues but in practice is difficult to establish.

The road transport sector is arguably a uniquely challenging sector for which to estimate emissions. In the UK alone, there are millions of individual vehicles that move in both space and time, representing a wide range of fuel types, emission standards, vehicle classes, and technologies. Even nominally identical vehicles may behave differently based on driver behavior, vehicle mileage, and levels of maintenance.^2,3^ Moreover, environmental conditions, such as the influence of ambient temperature, can also have an effect on road vehicle emissions.^4,5^

Of particular recent interest has been the emission of NO_*x*_ from road vehicles. Given the wide ranging impacts of NO_*x*_ emissions into the atmosphere, it is important that emission estimates are robust and representative of the region being considered. In Europe, over the past decade, there has been substantial focus on how road vehicle emissions of NO_*x*_ contribute to ambient nitrogen dioxide (NO_2_) concentrations, which have often exceeded ambient air quality limits.^6^ Emissions of NO_*x*_ also play a central role in the formation of O_3_ and PM_2.5_, both of which are important pollutants from a direct health impact perspective and in terms of wider environmental damage. Extensive evidence of considerable differences between emissions measured in the laboratory for Type Approval purposes and real driving emissions has also been widely reported and is well established.^7,8^ However, the incorporation of increasingly available real driving emissions data to emission inventories has not been as extensive.

In the UK, the National Atmospheric Emissions Inventory (NAEI) is the primary inventory that categorizes the emissions of many greenhouse gases and air quality pollutants. It covers multiple sectors, including industry, agriculture, land-use, energy generation, and transport.^9^ In 2018, the NAEI indicated that the transport sector was responsible for 52% of the UK’s NO_*x*_ emissions, with 31% coming from road transport.^10^ The NAEI forms the basis of reporting total UK emissions as a part of the National Emissions Ceiling Directive,^1^ as well as providing an input to local and regional scale air quality models. It is important therefore that the inventory accurately represents the emissions from sectors such as road transport.

Like many European emission inventories, the UK NAEI relies heavily on the COPERT (COmputer Program to calculate Emissions from Road Transport) emission factor approach for estimating road transport emissions,^11,12^ based on recommendations from the European Monitoring and Evaluation Program (EMEP)/European Environment Agency (EEA) Emission Inventory Guidebook.^13^ Initially, the emission factor development was based entirely on laboratory measurements. More recently, portable emission measurement systems (PEMSs) have been incorporated into the emission factor development. The 2019 EMEP/EEA guidebook notes that a combination of laboratory and on-board measurements are now typically used for emission factor development, with other methods such as vehicle emission remote sensing and tunnel studies being used for validation purposes. Indeed, the literature encompasses studies which have used PEMS,^14,15^ vehicle emission remote sensing,^16,17^ and even aircraft-based flux measurements^18^ to independently validate emission inventory estimates.

Measuring relatively few vehicles using laboratory-based or on-vehicle measurement techniques such as PEMS can provide detailed single vehicle emission information, but it is challenging to measure many vehicles using these methods due to cost and time constraints. It is known that emissions can vary significantly by the vehicle manufacturer and vehicle model, but currently no account is taken of these differences in the emission factor or inventory development.^7^ Choosing a representative sample of a country’s vehicle fleet from which to derive emission factors is therefore a potentially important issue. The advantage of remote sensing over other methods are the large sample sizes and comprehensive fleet coverage, which provides a better representation of in-use vehicle fleets.

A focus on the UK over other European countries for inventory verification is advantageous given that Great Britain is an island. In countries such as Germany, France, and Belgium, gasoline and diesel fuel sold may not be used within the country itself, leading to some uncertainty in the allocation of fuel use (and hence emissions) to a specific country. Conversely, in the UK close to 100% of road transport fuel sold is used in the UK. This means that robust comparisons can be made between so-called “bottom-up” and “top-down” inventory methods. Specifically, there is high certainty in the top-down calculations that rely on total fuel sale data.

The primary focus of this work is to exploit the comprehensive fleet coverage provided by vehicle emission remote sensing to develop highly detailed and comprehensive bottom-up NO_*x*_, CO, and NH_3_ emissions estimates at a UK scale for light-duty vehicles (LDVs). We achieve this aim through the calculation of distance-based emission factors and make direct comparisons with the 2018 UK inventory. Additionally, calculations are made of CO_2_ emissions to enable a direct comparison with fuel use statistics and provide a means of verifying the methods developed.

A specific focus is to estimate NO_*x*_ emissions, which have persistently been thought to be underestimated, and provide a national level quantification of total emissions. Finally, for the first time, we consider the influence of different vehicle manufacturer fleet mixes, which can be determined from remote sensing data. By considering different measured vehicle manufacturer proportions in other European countries, we establish how these contrasting manufacturer proportions affect total emissions of NO_*x*_ and CO_2_.

## 2 Materials and Methods

### 2.1 Vehicle Emission Remote Sensing

The development of and operating principles behind vehicle emission remote sensing has been described in considerable detail in other publications,^19,20^ but is summarized here. A remote sensing device (RSD) consists of a UV/IR source, multiple detectors, optical speed-acceleration bars, and a number plate camera. A RSD is deployed such that vehicles drive past the set-up unimpeded, with the concentrations of gases in their exhaust plumes and their speed and acceleration being measured remotely via open path spectroscopy. Spectrometry is achieved using a collinear beam of IR and UV light which, after being absorbed by exhaust plumes, is separated into its two components within the detector. Nondispersive infrared detectors measure CO, CO_2_, hydrocarbons (HCs), and a background reference. The UV component passes through a quartz fiber bundle and is used to measure NH_3_, NO, and NO_2_.

One hundred measurements are taken in half a second for each vehicle plume exhaust when the rear of the vehicle is detected. From these measurements, the ratio of a pollutant to CO_2_ is calculated, from which fuel-specific (g kg^–1^) emission factors can be calculated. The further transformation from fuel-specific to distance-specific (g km^–1^) emission factors is described later in the text.

Vehicle number plates are recorded alongside emission and speed measurements and are used to obtain vehicle technical data, such as engine size, fuel type, Euro standard, and vehicle manufacturer. In this study, the data were obtained from CDL Vehicle Information Services Ltd., a commercial supplier. CDL retrieved the data from the Driver and Vehicle Licensing Agency and the Society of Motor Manufacturers and Traders Motor Vehicle Registration Information System. Data relating to the total mileage of each vehicle at its last annual technical inspection test was also obtained through CDL for vehicles greater than three years old.

Vehicle emission measurements were conducted between 2017 and 2020 at 37 sites across 14 regions in the United Kingdom using two remote sensing instruments—the majority with the Opus AccuScan RSD 5000,^21^ supplemented with the data from the University of Denver Fuel Efficiency Automobile Test (FEAT) instrument.^22^ A total of 304,039 measurements were collected of Euro 2–6 vehicles in three key classes of LDVs: diesel light commercial vehicles (LCVs) and diesel and gasoline passenger cars (PCs). A statistical summary of the data set is provided in Table 1.

**Table 1:** Statistical Summary of the Vehicle Emission Remote Sensing Data, Split into Diesel LCVs and Diesel and Gasoline PC

characteristic	diesel LCV	diesel PC	gasoline PC
# of measurements	55,018	113,554	135,467
# of manufacturers	34	51	61
(with ≥100 measurements)	16	34	39
VSP^a^ (kW t^–1^)	5.1 (7.4)	6.3 (8.1)	5.9 (7.5)
speed^a^ (km h^–1^)	34.2 (10.1)	35.2 (10.1)	35.0 (9.9)
acceleration^a^ (km h^–1^ s–1)	0.99 (2.25)	1.16 (2.40)	1.02 (2.29)
temperature^a^ (°C)	13.9 (5.1)	14.9 (5.3)	14.9 (5.2)
mileage^a^ (1000 km)	169.2 (102.1)	147.2 (105.7)	112.3 (72.9)
Euro standard^b^			
Euro 2	290 (0.5%)	488 (0.4%)	3191 (2.4%)
Euro 3	3912 (7.1%)	9222 (8.1%)	23,272 (17%)
Euro 4	11,472 (21%)	22,743 (20%)	33,946 (25%)
Euro 5	27,985 (51%)	45,900 (40%)	39,691 (29%)
Euro 6	11,359 (21%)	35,201 (31%)	35,367 (26%)
RSD^b^			
Opus RSD 5000	47,140 (86%)	99,294 (87%)	118,379 (87%)
Denver FEAT	7878 (14%)	14,260 (13%)	17,088 (13%)

a Statistics presented: mean (standard deviation).

b Statistics presented: number of measurements (percentage of the column total).

### 2.2 Calculating Distance-Specific Emission Factors

The calculation of distance-specific (g km^–1^) emission factors is required for the “bottom-up” approach to estimating total UK emissions. The vehicle power-based approach used has been previously developed and evaluated^23,24^ but is briefly outlined here. The principal steps include (i) the development of a vehicle power-based method to calculate g km^–1^ emissions from remote sensing data, (ii) development of relationships that enable the prediction of emissions over any 1-Hz drive cycle, and (iii) the application of the g km^–1^ emissions to a UK national scale. Because vehicle emission remote sensing measurements tend to be made under higher engine load conditions than full drive cycle averages, their direct use would tend to overestimate mean exhaust emissions. The method provides a way in which to estimate emissions for typical real-world drive cycles that may have lower average engine loads, for example, for typical urban driving.

A physics-based approach to calculating vehicle power is used, accounting for all the main forces acting on a vehicle. First, instantaneous vehicle power is calculated as the total power to accelerate the vehicle, to overcome the road gradient, to resist both rolling and air resistance, and to power auxiliary devices adjusted for losses in the transmission. Vehicle specific power, VSP, is calculated as the instantaneous power divided by the vehicle mass (assumed to be the curb weight plus 150 kg to account for the weight of the driver, passengers, and cargo). As none of the road load or aerodynamic drag coefficients were known, generic values taken from Davison et al.^23^ were used. Fuel consumption is straightforwardly calculated from VSP using a linear model relating VSP to fuel consumption using the PC and Heavy Duty Emissions Model.^25^ As the parameters were based on Euro 5 and 6 vehicles, a 5% penalty was applied to Euro 2–4 vehicles to account for poorer fuel efficiency. Fuel-specific emission factors in g kg^–1^ can then be combined with fuel consumption in kg s^–1^ to produce time-specific emission factors (g s^–1^).

Relationships between emissions in g s^–1^ and VSP for vehicles with different fuel types, vehicle types, Euro standards, and pollutant species were established using generalized additive models (GAMs), which are flexible enough to consider nonlinear relationships between variables. The *mgcv* R package^26^ was used to fit the models. These models were used to predict emissions for 1 Hz drive cycles from PEMS tests obtained from the UK Department for Transport (DfT).^27^ The PEMS data contained a total of 4,243 km of real-world driving over 58 PEMS routes which included urban, rural, and motorway portions. The maximum VSP value across these drive cycles was 37.2 kW t^–1^ (equal to the 99.2% VSP value of the remote sensing measurements), and GAMs were fit between 0 and 40 kW t^–1^. Emissions from negative VSP conditions were assumed to be zero. The approach is flexible enough that it can be applied to any 1 Hz drive cycle, for which VSP is available or can be calculated.

With 1 Hz modeled time-specific emissions, distance-specific emission factors (g km^–1^) can be calculated as the total of all time-specific emissions divided by the total distance. The distance-specific emission factor used for the total UK emission estimation was the mean of all the distance-specific factors from each of the 58 real-world drive cycles. Factors were calculated separately for each of the urban, rural, and motorway conditions. The next step is to apply these emission factors to the corresponding driving activity data in the UK, thus providing a means of estimating total UK emissions.

### 2.3 Estimating Total UK Emissions

Distance-specific emission factors for each vehicle type were used to calculate a bottom-up estimate of total UK emissions through multiplication with UK-wide mileage data. Estimates of the total distance travelled by UK PCs and LCVs per annum were obtained from a publicly available government database.^28^ This activity data were obtained by the UK Department for Transport using a national network of around 180 automatic traffic counters, which used recorded physical properties of vehicles to segment these into vehicle types (PCs, vans, etc.). In order to apportion this vehicle mileage data into different fuel types, information available in the remote sensing data, such as average mileages by fuel type, was used, as provided in Table 1.

The vehicle mileages are already apportioned into urban, rural, and motorway driving conditions but not by fuel type or Euro standard. The data in Table 1 indicate that there is a 1:1.32 ratio of recorded mileage between gasoline and diesel PCs, but a 1.11:1 ratio of number of measurements. The number of measurements provides a direct measure of vehicle km driven under urban conditions given where remote sensing measurements are made. In other words, diesel vehicles drive further on an overall UK level compared with gasoline vehicles, but gasoline vehicles drive further than diesel vehicles in urban areas. The rural and motorway portions were adjusted proportionally such that the sum of the urban, rural, and motorway portions summed to the total annual mileage reported in UK statistics. Only 0.71% of LCVs measured were gasoline, which have not been explicitly considered given their low numbers and minor contribution to emissions. However, overall LCV mileage data were reduced by this small amount to apply to diesel LCVs only.

Apportionment into Euro standards is straightforward, simply applying the ratio between the five Euro standards for each of the three vehicle categories—Diesel PC, Gasoline PC, and Diesel LCV—given in Table 1. The fully apportioned mileages are provided in Table S1. To calculate UK totals for the exhaust pollutants, the g km^–1^ emission factors for each combination of pollutant species, vehicle category, Euro standard, and driving condition (urban, rural or motorway) were multiplied by the corresponding apportioned mileage. While emission inventories themselves are often not reported with the associated uncertainties, the estimates presented here are provided alongside the 95% confidence interval calculated from the original g kg^–1^ measurements.

The estimated UK totals can be directly compared with the NAEI. The comparison can be expressed through the use of a ratio between the bottom-up estimated emission and the emission reported in the NAEI, here labeled *F*. The value of *F* is therefore also the factor by which one would multiply the emission reported in the NAEI to arrive at the emission estimated using the vehicle emission remote sensing data. A *F* of 1 would mean that these two values were the same, *F* > 1 would mean the emission is under-reported in the NAEI and *F* < 1 would mean that the emission is over-reported.

The NAEI reports air quality pollutant sources for *four* driving conditions—urban, rural, and motorway, and a separate cold start contribution. In common with most emission inventories, the increased emissions of some pollutants after engine start are considered as separate emissions from hot, stabilized emissions. For some pollutants, such as CO and HCs, the cold start emissions can be substantial. In the NAEI, cold start emissions are only considered in urban areas and reflect the estimated number of trips.

The potential importance of cold start emissions raises the question about the extent to which vehicle emission remote sensing includes a cold start contribution. Given that the vast majority of emission measurements are made in urban areas, it might be expected that remote sensing data would include some fraction of elevated emissions due to cold starts. However, for gasoline vehicles, the three-way catalyst reaches effective operating temperature (called “light-off”) within 1–2 min of the engine starting.^29^ This means that it is highly unlikely that remote sensing measurements include a significant proportion of cold start emissions given the proximity required of a cold start to the measurement location. Therefore, when urban comparisons are made, the estimates are compared with both the urban value from the NAEI and a combination of the urban and cold start contributions.

The NAEI is required to report road transport emissions of CO_2_ from fossil fuels only, so the figures reported do not include the additional presence of biofuels. Assuming that diesel in the UK contains up to 3.7% biodiesel and gasoline up to 4.6% bioethanol,^30^ an adjustment factor can be calculated through the multiplication of the bio-/fossil-fuel ratio by the ratio of fuel CO_2_ emissions (kg) per liter of the biofuel and fossil fuel (1.52/2.31 for gasoline, 2.36/2.69 for diesel).^31^ The adjustments are therefore 1.032 for gasoline and 1.034 for diesel and used to uplift the reported NAEI CO_2_ values.

### 2.4 Effects of the Vehicle Fleet Composition

To investigate the importance of different fleet compositions in European countries, data from the CONOX project were analyzed, which provides a database of European vehicle emission remote sensing measurements.^32^ These data provide over 700,000 remote sensing measurements for the UK, Sweden, Switzerland, Belgium, France, and Spain. The data usefully contain information on the breakdown of different manufacturers and vehicle models, which can be used to consider the effects on NO_*x*_ emissions due to different national fleet mixes. An advantage of these data is that they provide a direct, on-road measurement of the vehicle fleet, which accounts for the vehicle km driven by vehicles made by different manufacturers. These data are considered more representative of in-use vehicle fleets than, for example, statistics on new vehicle sales, which would not reflect actual distances travelled by different vehicle types. The data do show strong country-specific characteristics. For example, France is dominated by Renault and Peugeot-Citroen, Sweden by Volkswagen and Volvo, and Switzerland by Volkswagen and, to a lesser extent, Daimler and BMW (Figure S1).

We have considered the total emissions of CO_2_ and NO_*x*_ based on UK mileage data for Euro 5 and Euro 6 diesel PCs but using the fleet mix for each country. In this respect, the analysis addresses the question of “how would UK emissions of NO_*x*_ change if the UK had the fleet of France, Spain, Belgium, Switzerland, or Sweden?” The calculations keep the vehicle km the same between the fuel type used and Euro standard, that is, that of the UK, and simply considers different proportions of manufacturer families according to the fleets in other countries. Manufacturer and engine size-specific emission factors were developed for this purpose using the UK-based data set outlined in the Vehicle Emission Remote Sensing subsection, using the same method as outlined in the Calculating Distance-Specific Emission Factors subsection.

## 3 Discussion

### 3.1 Total UK LDV Emissions

**Figure 1:**
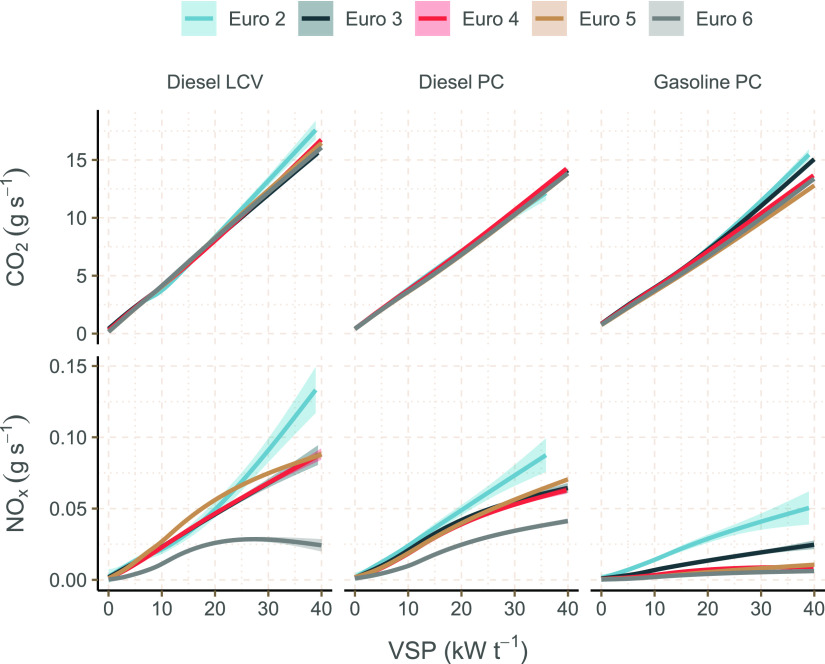
GAMs fit using data from vehicle emission remote sensing relating vehicle CO_2_ and NO_*x*_ g s^–1^ to VSP, colored by Euro classification and faceted into three LDV categories. The shading shows the standard error of the GAM fit.

The relationship between VSP and emission rate in g s^–1^ for NO_*x*_ and CO_2_ is shown in Figure 1, based on the GAMs developed from the vehicle emission remote sensing data for each fuel type, vehicle type, and Euro standard. ANOVA testing of fitted GAMs confirmed the significance (*P* < 0.05) of VSP in modeling both CO_2_ and NO_*x*_ in all three vehicle categories for all five Euro standards considered. Most of the relationships shown in Figure 1 are close to linear; particularly for CO_2_, which highlights the benefit of expressing emissions as a function of vehicle power demand rather than vehicle speed. Indeed, an inherent problem with speed-dependent emission factors is that as the speed tends to zero, the emissions tend to infinity, which means fitting a model through the data is difficult.

All predicted CO_2_ and NO_*x*_ emissions and their associated *F* values are tabulated in Table 2. Key values and implications are described here.

**Table 2:** Bottom-Up Vehicle Emission Remote Sensing CO_2_ and NO_*x*_ Predictions for Different Vehicle Categories and Driving Conditions, with Associated *F* Values^a^

		carbon dioxide/CO_2_		nitrogen oxides/NO*x*	
vehicle category	driving conditions	prediction (Mt)	F	prediction (kt)	F
all LDVs	all	91.3 ± 0.9	1.01	280 ± 6.3	1.24–1.32
	urban	40.3 ± 0.4	1.17	103 ± 2.4	1.22–1.47
	rural	34.6 ± 0.3	0.92	115 ± 2.5	1.27
	motorway	16.4 ± 0.2	0.93	62.6 ± 1.3	1.21
gasoline PCs	all	35.2 ± 0.30	1.00	29.5 ± 1.5	1.82–1.95
	urban	19.3 ± 0.2	1.23	15.0 ± 0.7	1.94–2.24
	rural	11.9 ± 0.1	0.84	10.7 ± 0.5	1.71
	motorway	4.01 ± 0.03	0.75	3.81 ± 0.2	1.77
diesel LDVs	all	56.1 ± 0.61	1.02	251 ± 5.0	1.19–1.27
	urban	21.1 ± 0.2	1.12	87.8 ± 1.7	1.15–1.38
	rural	22.6 ± 0.2	0.96	104 ± 2.0	1.24
	motorway	12.4 ± 0.1	1.01	58.8 ± 1.1	1.18
diesel PCs	all	40.4 ± 0.4	1.14	169 ± 2.9	1.44–1.54
	urban	15.0 ± 1.2	1.22	57.7 ± 1.5	1.22–1.46
	rural	16.1 ± 1.1	1.07	70.0 ± 1.6	1.55
	motorway	9.21 ± 1.1	1.15	41.7 ± 1.6	1.64
diesel LCVs	all	15.7 ± 0.2	0.81	81.2 ± 2.0	0.88–0.94
	urban	5.99 ± 0.09	0.92	30.2 ± 0.7	1.03–1.26
	rural	6.48 ± 0.10	0.76	34.0 ± 0.8	0.88
	motorway	3.20 ± 0.05	0.74	17.0 ± 0.4	0.70

a The urban and total driving conditions are given as a range, reflecting both hot urban emissions from the NAEI and a combination of hot urban and cold start emissions.

An important first step is to establish whether there is a carbon/energy balance for the detailed bottom-up approach to estimate CO_2_ at a national scale. The total estimated emissions from this method were 91.3 ± 0.9 Mt CO_2_. This value is very similar to the NAEI value of 90.0 Mt, giving an *F* value equal to 1.01. The similarity extends when considering the two fuel types independently—gasoline vehicles were shown to have an *F* value of 1.00 and diesel vehicles 1.02. When considering diesel PCs and LCVs separately; however, divergence from the NAEI is apparent, with the PCs having an associated *F* of 1.14 and the LCVs 0.81. The bottom-up calculations therefore suggest a different allocation of diesel fuel use (or CO_2_ emissions) than is suggested by the NAEI, although the sum of PC and LCV CO_2_ is in good agreement. It should be noted that the comparison for gasoline is considered more robust than for diesel fuel because almost all gasoline use in the UK (97%) is for PCs, whereas diesel fuel is used in a wide range of vehicle types including PCs, LCVs, buses, and other heavy-duty vehicles, which introduces some uncertainty in the allocation between diesel-fueled vehicles.^33^

With respect to NO_*x*_, the total UK estimates were 280 ± 6.3 kt NO_*x*_. On a UK scale, the NAEI underestimates NO_*x*_ emissions, with *F* between 1.24 and 1.32 depending on whether cold start emissions are included or excluded, respectively. These comparisons can be made at a more disaggregated level by considering the vehicle categories individually. Estimated gasoline PC emissions were higher than those reported in the NAEI, with NO_*x*_ emissions of 29.5 ± 1.5 kt (1.82 < *F* < 1.95). The NO_*x*_ predictions for light-duty diesel vehicles were similarly under-reported in the NAEI, being 251 ± 5.0 kt NO_*x*_ (1.19 < *F* < 1.27). Of this diesel total, PCs contribute 169 ± 2.9 kt NO_*x*_ (1.44 < *F* < 1.54) and LCVs 81.2 ± 2.0 kt NO_*x*_ (0.88 < *F* < 0.94).

**Figure 2:**
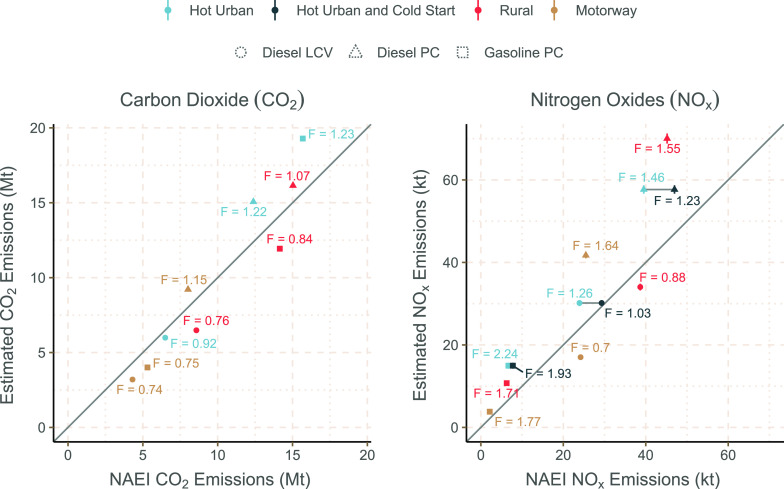
Total UK estimates for CO_2_ and NO_*x*_ using vehicle emission remote sensing, in comparison with the 2018 emissions reported in the national inventory. *F* values, representing the ratio between the bottom-up estimate and the reported NAEI value, are provided. Urban bottom-up estimates are compared with both hot urban emissions from the NAEI and a combination of hot urban and cold start emissions, shown connected by a grey horizontal line. Error bars show the 95% confidence intervals projected from the fuel-specific (g kg^–1^) emission factors. The grey diagonal line shows a 1:1 relationship.

The comparison between the NAEI and the bottom-up remote sensing data estimations is made on a fully disaggregate level, including vehicle category and driving condition, as shown in Figure 2. This analysis shows broad consistency between the bottom-up estimates and NAEI reported values for CO_2_, with *F* values between 0.77 and 1.27. Conversely, NO_*x*_ is shown to have *F* values between 0.70 and 2.24, with some important variability depending on driving conditions (urban, rural, or motorway).

A specific interest is the quantification of NO_*x*_ emissions in urban areas where exposures to the elevated concentrations of NO_2_ are the greatest. In total, the NAEI reports 84.0 kt NO_*x*_ from LDV activity in urban areas and from cold start emissions, with 70.1 kt coming from just urban emissions. Conversely, the new bottom-up estimates suggest total urban NO_*x*_ emissions of 103 ± 2.5 kt, a difference of 19 kt including cold start emissions or 32.9 kt excluding them. These results suggest the NAEI may be under-reporting urban emissions by 22–47%. As discussed previously, it is considered that the remote sensing measurements comprise a very low proportion of enhanced emissions due to cold start effects. For this reason, the underestimate in urban NO_*x*_ emissions is considered to be closer to 47% than 22%.

The total UK bottom-up estimates for the other air quality pollutants were 537 ± 25.4 kt CO and 9.1 kt ± 0.5 NH_3_. At the UK scale, the NAEI is seen to consistently underestimate these emissions, with *F* = 2.86 for CO and *F* = 2.23 for NH_3_. The equivalent visualization, as shown in Figure 2, including these additional pollutants is provided as Figure S3.

It is important to consider the underlying reasons behind the disparity between the bottom-up estimates and the values reported in the NAEI, which could be associated with vehicle fleet assumptions and/or the emission factors. We have re-calculated the bottom-up emissions based on the fleet composition assumptions used in the NAEI^34,35^ and the NAEI allocations of gasoline and diesel fuel use in urban areas. The NAEI assumed a newer vehicle fleet compared with the observation-based values used for the bottom-up calculations. Using these NAEI assumptions resulted in UK-wide LDV emissions with *F* values of 1.05 for CO_2_ and 1.06–1.13 for NO_*x*_, or 1.19 and 1.05–1.26 in only urban areas. However, there were some significant disparities on a disaggregated level when using NAEI fleet assumptions, for example, with *F* = 1.20 for gasoline CO_2_ (compared with *F* = 1.00 using the bottom-up methods). These results strongly suggest that the use of the observation-based fleet information in the bottom-up emission calculations provide a much better explanation of the total UK emissions. On this basis, much of the discrepancy between the NAEI and the bottom-up methods is associated with the vehicle fleet and vehicle activity assumptions rather than the emission factors. Nevertheless, even adopting the NAEI vehicle fleet assumptions still results in up to a 26% underestimation of NO_*x*_ emissions compared with the bottom-up calculation in urban areas.

### 3.2 Influence of the Vehicle Fleet Composition

An inherent benefit of the vehicle emission remote sensing data for use in the emission factor and emission inventory development is the comprehensive coverage of a wide range of vehicle manufacturers and models, which is difficult to achieve through laboratory or PEMS studies owing to the large number of vehicles that would need to be tested. Vehicle fleets can vary from smaller city-wide to larger country-wide scales. For example, some cities may tend to have a higher than average proportion of vehicles from a certain manufacturer (e.g., taxis or local government vehicles).

**Figure 3:**
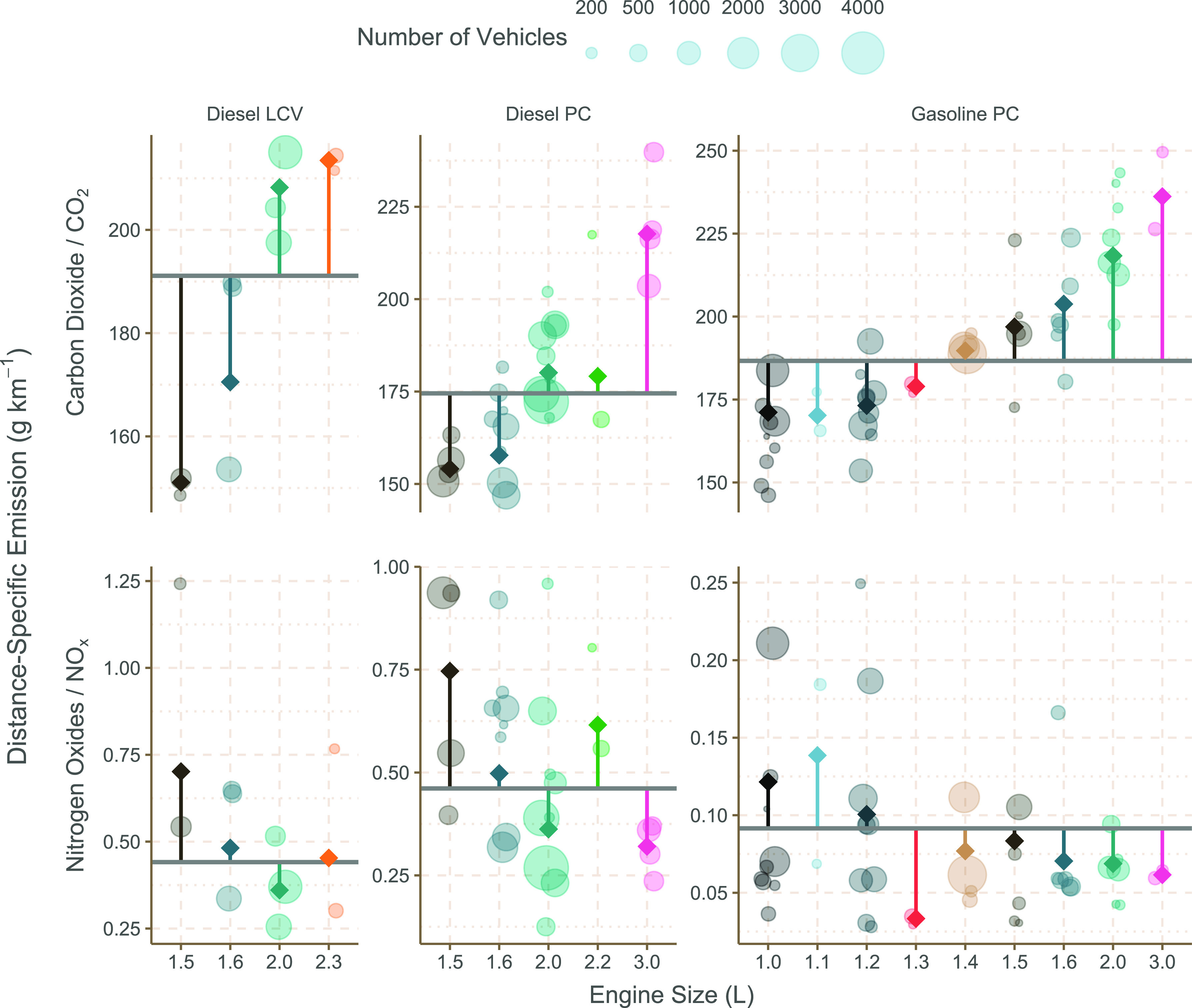
Distance-specific CO_2_ and NO_*x*_ emissions (g km^–1^) for Euro 6 LDVs. Each dot represents a unique manufacturer group-engine size combination, with a size proportional to the number of observations included in its calculation. The diamonds represent the weighted mean for each engine size, and the horizontal lines the weighted mean for each vehicle category (diesel LCV, diesel PC, gasoline PC).

Figure 3 provides an example of the variation in NO_*x*_ emissions between different manufacturer groups and engine sizes, revealing the considerable differences from the mean levels of emissions for each engine size (visualized as diamonds) and vehicle category (horizontal lines). In this case, manufacturer “families” have been used, which groups similar engine types across different manufacturers.^7^ For example, the Volkswagen group (VWG) consists of Volkswagen, Audi, Skoda, and Seat. With large databases of vehicle emission remote sensing data, it is possible to disaggregate the data further. For example, an account can be taken of the mandatory and voluntary software and hardware fixes applied to certain VWG vehicles following the diesel gate scandal, which has had an appreciable effect on reducing NO_*x*_ emissions from certain vehicle models; reducing emissions between 30 to 36%.^36^

Emission factor models used throughout Europe do not account for manufacturer-level differences in emissions and instead provide generic factors, for example, for Euro 5 diesel PCs below 2.0 L engine capacity. However, it is clear from Figure 3 that there can be large differences in emissions of NO_*x*_ between different manufacturers and vehicle models. Such differences would not be important if vehicle fleets were uniformly mixed throughout Europe. However, there are considerable differences between the compositions of vehicle fleets across different countries, which could have important effects on country-level emissions of different pollutants.

**Figure 4:**
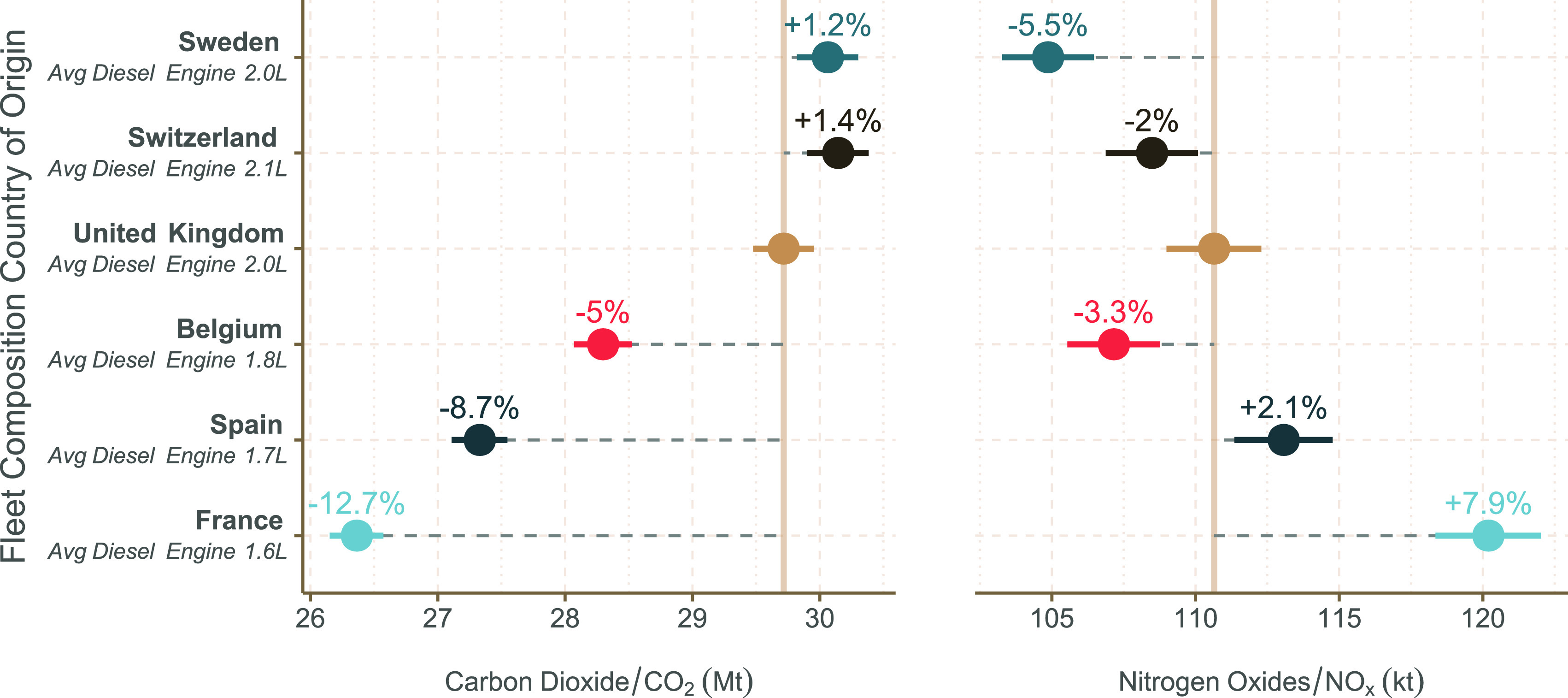
Total CO_2_ and NO_*x*_ emissions from Euro 5 & 6 diesel PCs using UK activity data and the relative fleet composition of the UK and five other European countries. Estimations were made using the manufacturer group and engine size-specific distance-based emission factors. Each of the non-UK fleet compositions are shown relative to the UK fleet. The error bars correspond to the 95% confidence interval. Also provided are the average Euro 5 & 6 diesel car engine size.

The results of the fleet composition analysis are shown in Figure 4 and demonstrate the impact of considering manufacturer-specific emissions representative of fleets in other countries. For example, estimates of NO_*x*_ from a French-like fleet of diesel cars are 7.9% higher than a UK-like fleet, despite the fact that CO_2_ emission estimates decrease by 12.7%. Conversely, the NO_*x*_ estimate of a Swedish fleet mix is 5.5% lower despite a 1.2% increase in CO_2_.

In general, Figure 4 highlights an overall trade-off at a country fleet level between CO_2_ and NO_*x*_ in that as CO_2_ emissions decrease, emissions of NO_*x*_ tend to increase. The higher emissions of NO_*x*_ for a French fleet is attributable to two main factors. First, a higher proportion of small diesel-engine PCs, which tend to have higher NO_*x*_ emissions (see Figure 3). The average diesel PC engine size in the French fleet is 1695 cm^3^ compared with 2152 cm^3^ in Switzerland in the CONOX database. Larger diesel-engine vehicles tend to use selective catalytic reduction for NO_*x*_ control, which is highly effective, rather than Lean NO_*x*_ Traps that are not as effective for NO_*x*_ control.^37^ Second, France has a higher proportion of manufacturers such as Renault that tend to have higher in-use emissions of NO_*x*_ compared with most other manufacturers.^7^

Differences in the magnitude of NO_*x*_ emissions between the fleet of different countries, as shown in Figure 4, are potentially of significant importance at a national scale. There is, for example, a difference of 13.4% in calculated NO_*x*_ emissions between the Euro 5 & 6 diesel PC fleet of Sweden compared with that of France; differences that are not currently reflected in emission factors or inventories. This finding highlights the potential benefits of considering the fine details of vehicle fleets when attempting to estimate emissions. Given the growing amount of the detailed vehicle emission remote sensing data available in Europe and elsewhere,^7,38−41^ the methods adopted in the current work could be used in many other countries.

Furthermore, at a country level, increases or decreases in total NO_*x*_ emissions from current assumptions will likely have several implications. First, it would directly affect the evaluation of urban exposures to concentrations of NO_2_, with potential impacts on meeting European Directive annual mean limits of 40 μg m^–3^. Second, a country-level change in estimated NO_*x*_ emissions of around 10% compared with the current assumptions would have wider air quality implications; especially for regional air quality modeling activities.

## Supporting Information Available

Treemaps showing the ten most popular manufacturer groups for Euro 5 and 6 diesel PCs in the five European countries contained within the CONOX remote sensing database, GAMs relating PC CO_2_, NO_*x*_, and CO g s^–1^ and NH_3_ mg s^–1^ to VSP, colored by Euro classification and faceted into three LDV categories, total UK estimates for CO_2_, NO_*x*_, CO, and NH_3_ using vehicle emission remote sensing, in comparison with the 2018 emissions reported in the National Atmospheric Emissions Inventory, annual UK PC and LCV mileage in billions of kilometers, rounded to two decimal places, and disaggregated based on driving conditions and Euro standard (ES), distance-based emission factors in g km^–1^ for the carbon containing species, CO_2_ and CO, and distance-based emission factors in g km^–1^ and mg km^–1^ for the nitrogen containing species, NO_*x*_ and NH_3_ (PDF)
