# Supplementary material for: Verification of a National Emission Inventory and Influence of On-road Vehicle Manufacturer-Level Emissions
Source: Environ Sci Technol. 2021 Mar 19;55(8):4452–61. doi: 10.1021/acs.est.0c08363 (PMC8154370; doi:10.1021/acs.est.0c08363)
Supplement: Supplementary file 1 — es0c08363_si_001.pdf [file es0c08363_si_001.pdf]

Supporting Information: Verification of a national emission inventory and influence of on-road vehicle manufacturer-level emissions

Jack Davison,[*,†] Rebecca A. Rose,[‡] Naomi J. Farren,[†] Rebecca L. Wagner,[†] Tim P. Murrells,[‡] and David C. Carslaw[*,†,‡]

†*Wolfson Atmospheric Chemistry Laboratories, University of York, York, YO10 5DD, United Kingdom*

‡*Ricardo Energy & Environment, Harwell, Oxfordshire, OX11 0QR, United Kingdom*

E-mail: jd1184@york.ac.uk; david.carslaw@york.ac.uk

Number of Pages: 6

Number of Figures: 3

Number of Tables: 3

List of Figures

S1 Treemaps showing the eight most popular manufacturer groups for Euro 5 and 6 diesel passenger cars in the six European countries contained within the CONOX remote sensing database. The area of each rectangle in relation to the overall square reflects the share of the fleet that the corresponding manufacturing group represents. Manufacturers are divided into engine sizes, labelled in cubic centimeters. S4

S2 Generalised Additive Models (GAMs) relating passenger car CO_2, NO_x and CO $g\,s^{-1}$ and NH_3 $mg\,s^{-1}$ to VSP, coloured by Euro classification and faceted into three light duty vehicle categories. The shading shows the standard error of the GAM fit. S5

S3 Total UK estimates for CO_2, NO_x, CO and NH_3 using vehicle emission remote sensing, in comparison with the 2018 emissions reported in the National Atmospheric Emissions Inventory. F values, representing the ratio between the VERS estimate and the reported NAEI value, are provided. Urban VERS estimates are compared with both hot urban emissions from the NAEI and a combination of hot urban and cold-start emissions, shown connected by a grey horizontal line. Error bars show the 95% confidence intervals projected from the fuel-specific ($g\,kg^{-1}$) emission factors. The grey diagonal line shows a 1:1 relationship. S6

List of Tables

S1 Annual UK passenger car (PC) and light commercial vehicle (LCV) mileage in billions of kilometers, rounded to two decimal places and dissagregated based on driving conditions and Euro Standard (ES). The complete totals for each light-duty vehicle type (roughly 410 bn & 82 bn km for passenger cars and light commercial vehciles, respectively) is taken from Department for Transport quarterly traffic estimates (TRA25). Apportionment is based on fleet composition information obtained during vehicle emission remote sensing campaigns. S7

S2 Distance-based emission factors in $\mathrm{g\,km^{-1}}$ for the carbon containing species, CO_2 and CO. In this case "ES" refers to the Euro Status of the vehicle. The *Low.* and *High.* values represent the 95% confidence interval. S8

S3 Distance-based emission factors in $\mathrm{g\,km^{-1}}$ and $\mathrm{mg\,km^{-1}}$ for the nitrogen containing species, NO_x and NH_3. In this case "ES" refers to the Euro Status of the vehicle. The *Low.* and *High.* values represent the 95% confidence interval. S9

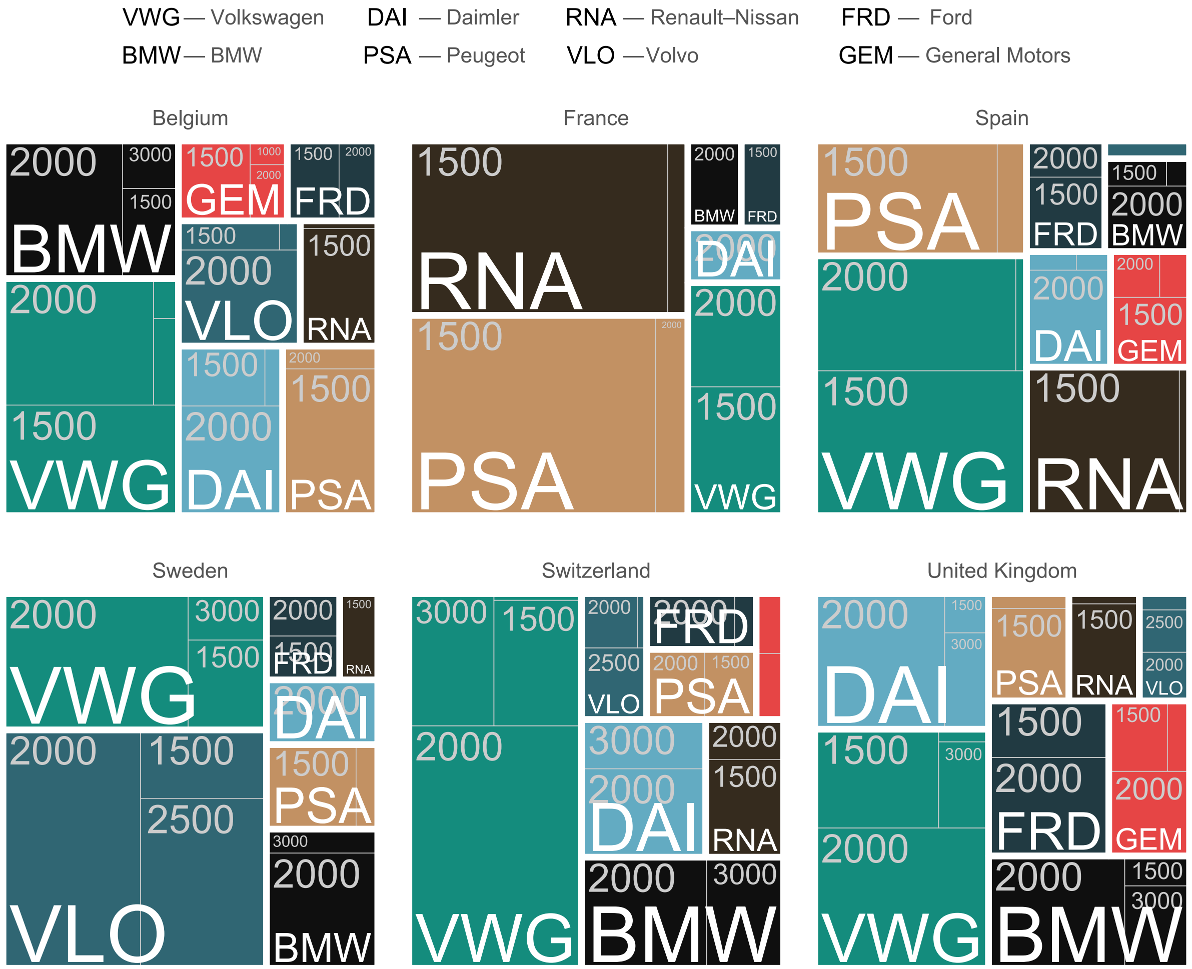

Figure S1: Treemaps showing the eight most popular manufacturer groups for Euro 5 and 6 diesel passenger cars in the six European countries contained within the CONOX remote sensing database. The area of each rectangle in relation to the overall square reflects the share of the fleet that the corresponding manufacturing group represents. Manufacturers are divided into engine sizes, labelled in cubic centimeters.

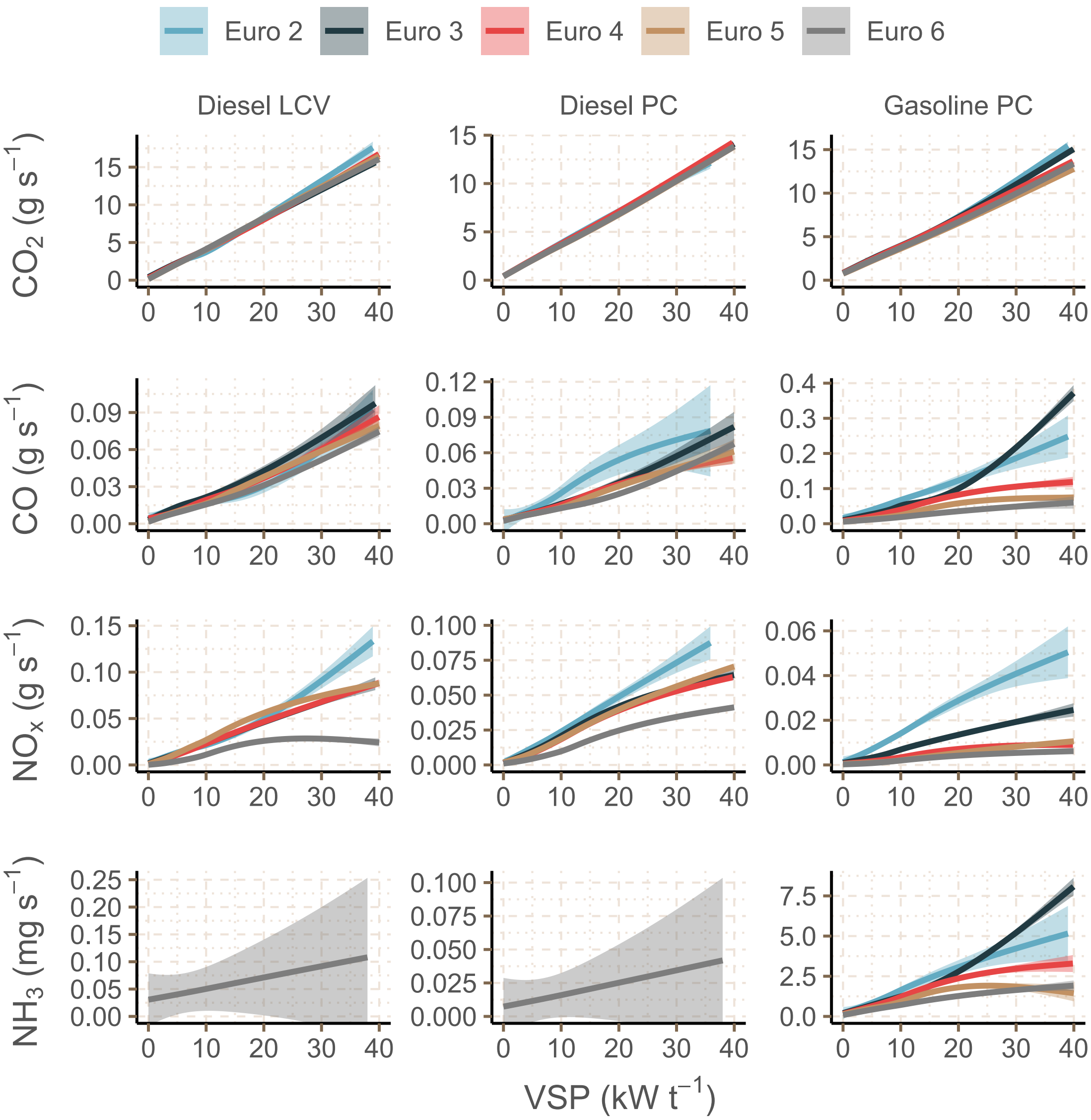

Figure S2: Generalised Additive Models (GAMs) relating passenger car CO_2, NO_x and CO $g\,s^{-1}$ and NH_3 $mg\,s^{-1}$ to VSP, coloured by Euro classification and faceted into three light duty vehicle categories. The shading shows the standard error of the GAM fit.

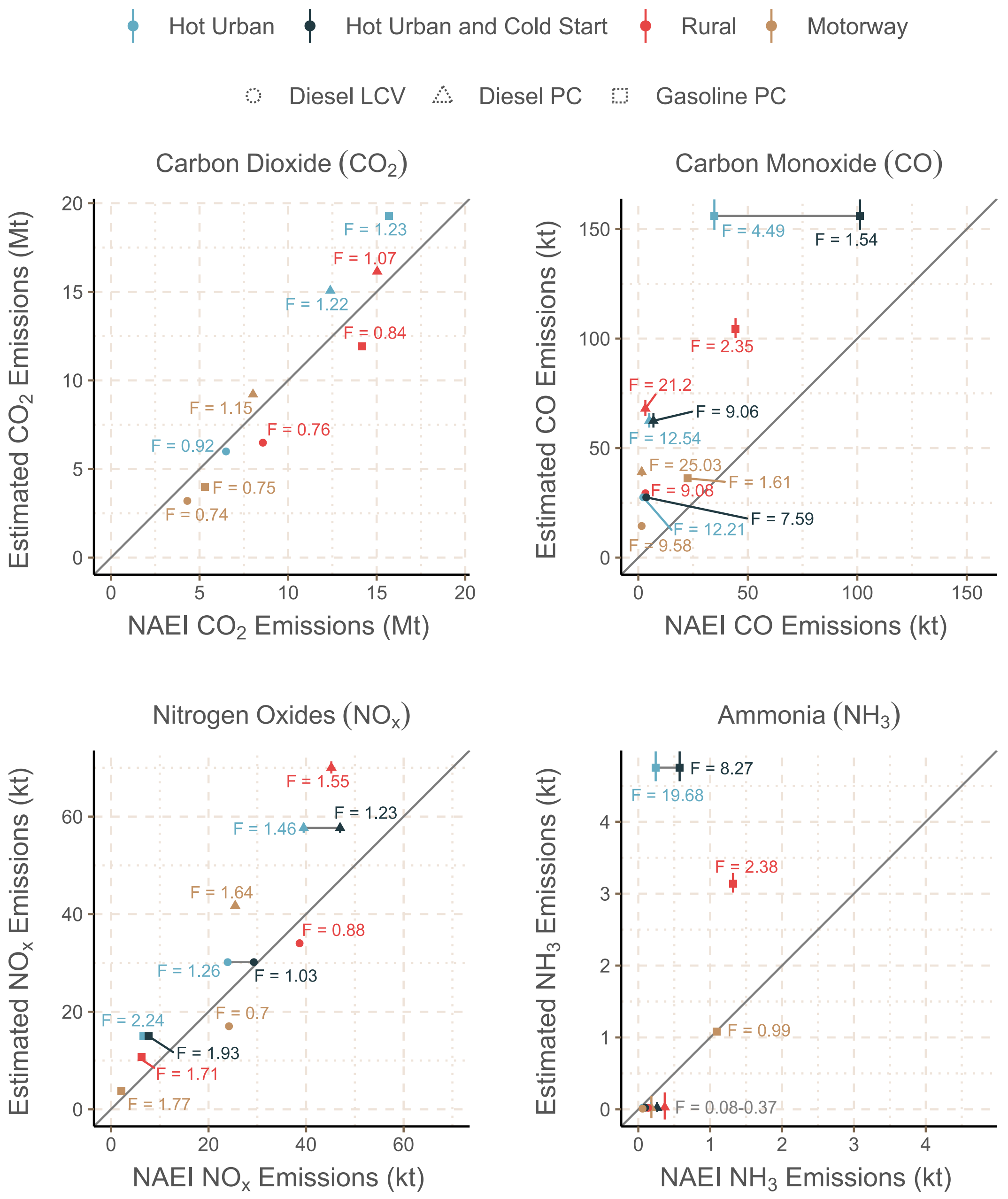

Figure S3: Total UK estimates for CO_2, NO_x, CO and NH_3 using vehicle emission remote sensing, in comparison with the 2018 emissions reported in the National Atmospheric Emissions Inventory. F values, representing the ratio between the VERS estimate and the reported NAEI value, are provided. Urban VERS estimates are compared with both hot urban emissions from the NAEI and a combination of hot urban and cold-start emissions, shown connected by a grey horizontal line. Error bars show the 95% confidence intervals projected from the fuel-specific ($\mathrm{g\,kg^{-1}}$) emission factors. The grey diagonal line shows a 1:1 relationship.

Table S1: Annual UK passenger car (PC) and light commercial vehicle (LCV) mileage in billions of kilometers, rounded to two decimal places and dissagregated based on driving conditions and Euro Standard (ES). The complete totals for each light-duty vehicle type (roughly 410 bn & 82 bn km for passenger cars and light commercial vehciles, respectively) is taken from Department for Transport quarterly traffic estimates (TRA25). Apportionment is based on fleet composition information obtained during vehicle emission remote sensing campaigns.

		**Annual UK Mileage (billion km)**			
**Category**	**ES**	**Urban**	**Rural**	**Motorway**	**Total**
Gasoline PC	2	1.89	1.78	0.57	4.24
	3	13.41	12.61	4.03	30.05
	4	19.72	18.54	5.93	44.19
	5	22.88	21.51	6.88	51.26
	6	20.51	19.28	6.17	45.96
	All	78.41	73.72	23.59	175.71
Diesel PC	2	0.28	0.43	0.22	0.93
	3	5.76	8.62	4.53	18.91
	4	14.22	21.28	11.19	46.68
	5	28.43	42.55	22.38	93.36
	6	22.03	32.98	17.34	72.36
	All	70.72	105.85	55.67	232.24
Diesel LCV	2	0.13	0.19	0.08	0.41
	3	1.90	2.69	1.19	5.78
	4	5.63	7.95	3.52	17.10
	5	13.68	19.30	8.56	41.53
	6	5.63	7.95	3.52	17.10
	All	26.98	38.07	16.89	81.93

Table S2: Distance-based emission factors in $g\,km^{-1}$ for the carbon containing species, CO_2 and CO. In this case "ES" refers to the Euro Status of the vehicle. The *Low.* and *High.* values represent the 95% confidence interval.

		**Urban**			**Rural**			**Motorway**		
	**ES**	**Low.**	**Avg.**	**High.**	**Low.**	**Avg.**	**High.**	**Low.**	**Avg.**	**High.**
**Carbon Dioxide (CO_2, $g\,km^{-1}$)**										
Gasoline PC	2	244.5	251.6	258.8	163.8	168.6	173.4	173.1	178.2	183.3
	3	257.2	259.8	262.3	169.3	171.0	172.7	177.6	179.4	181.1
	4	252.8	254.7	256.5	166.0	167.2	168.4	173.9	175.2	176.4
	5	232.6	234.2	235.9	153.2	154.2	155.3	160.8	161.9	163.0
	6	239.2	241.1	242.9	157.4	158.6	159.8	165.1	166.4	167.7
Diesel PC	2	202.9	219.8	238.8	144.9	157.1	170.6	157.1	170.2	184.9
	3	212.2	216.2	220.1	152.4	155.2	158.0	165.5	168.6	171.6
	4	217.0	219.1	221.4	155.4	157.0	158.6	168.7	170.3	172.1
	5	208.6	210.1	211.6	149.3	150.4	151.5	162.0	163.1	164.3
	6	210.0	211.8	213.7	150.3	151.6	152.9	163.0	164.4	165.9
Diesel LCV	2	206.4	228.8	255.7	156.6	173.6	194.1	173.5	192.4	215.0
	3	231.4	237.8	244.6	168.7	173.3	178.3	184.2	189.2	194.7
	4	224.6	228.4	232.5	168.5	171.3	174.4	185.9	189.1	192.5
	5	215.5	218.0	220.5	167.7	169.7	171.6	187.5	189.6	191.8
	6	216.0	220.2	224.3	166.6	169.8	173.0	185.6	189.2	192.8
**Carbon Monoxide (CO, $g\,km^{-1}$)**										
Gasoline PC	2	3.94	4.30	4.73	2.63	2.87	3.16	2.78	3.03	3.34
	3	2.77	2.88	2.99	2.15	2.23	2.32	2.40	2.49	2.59
	4	2.33	2.40	2.48	1.64	1.69	1.74	1.77	1.82	1.88
	5	1.50	1.55	1.60	1.08	1.11	1.15	1.17	1.20	1.24
	6	1.21	1.29	1.44	0.78	0.83	0.92	0.81	0.86	0.96
Diesel PC	2	1.03	1.34	1.69	0.81	1.05	1.33	0.91	1.18	1.49
	3	0.87	0.97	1.05	0.66	0.74	0.80	0.74	0.82	0.88
	4	0.94	0.98	1.01	0.67	0.69	0.72	0.72	0.75	0.77
	5	0.81	0.87	0.94	0.60	0.64	0.69	0.66	0.70	0.75
	6	0.79	0.82	0.85	0.57	0.59	0.61	0.62	0.64	0.66
Diesel LCV	2	1.11	1.29	1.47	0.73	0.85	0.97	0.77	0.89	1.02
	3	1.28	1.38	1.49	0.90	0.97	1.05	0.98	1.05	1.13
	4	1.05	1.10	1.17	0.77	0.81	0.86	0.85	0.89	0.94
	5	0.95	0.98	1.01	0.74	0.76	0.79	0.83	0.85	0.88
	6	0.88	0.91	0.95	0.66	0.69	0.71	0.73	0.76	0.78

Table S3: Distance-based emission factors in $\mathrm{g\,km^{-1}}$ and $\mathrm{mg\,km^{-1}}$ for the nitrogen containing species, NO_x and NH_3. In this case "ES" refers to the Euro Status of the vehicle. The *Low.* and *High.* values represent the 95% confidence interval.

		**Urban**			**Rural**			**Motorway**		
	**ES**	**Low.**	**Avg.**	**High.**	**Low.**	**Avg.**	**High.**	**Low.**	**Avg.**	**High.**
**Nitrogen Oxides (NO_x, $\mathrm{g\,km^{-1}}$)**										
Gasoline PC	2	0.70	0.77	0.84	0.53	0.58	0.63	0.59	0.65	0.70
	3	0.36	0.37	0.39	0.27	0.28	0.29	0.29	0.31	0.32
	4	0.18	0.19	0.20	0.13	0.14	0.15	0.15	0.15	0.16
	5	0.11	0.12	0.12	0.09	0.10	0.10	0.10	0.11	0.11
	6	0.10	0.10	0.11	0.08	0.08	0.08	0.08	0.09	0.09
Diesel PC	2	1.15	1.29	1.44	0.89	1.00	1.11	0.99	1.11	1.24
	3	1.06	1.09	1.12	0.81	0.83	0.85	0.89	0.92	0.94
	4	0.91	0.92	0.94	0.73	0.74	0.76	0.82	0.84	0.85
	5	0.94	0.95	0.96	0.76	0.77	0.78	0.85	0.87	0.88
	6	0.48	0.49	0.50	0.42	0.43	0.44	0.48	0.49	0.51
Diesel LCV	2	1.12	1.31	1.54	0.91	1.06	1.24	1.02	1.20	1.40
	3	1.22	1.27	1.33	0.92	0.96	1.00	1.01	1.06	1.11
	4	1.12	1.15	1.18	0.90	0.92	0.95	1.01	1.04	1.07
	5	1.32	1.34	1.36	1.05	1.07	1.09	1.18	1.20	1.22
	6	0.46	0.48	0.51	0.39	0.42	0.44	0.45	0.48	0.51
**Ammonia (NH_3, $\mathrm{mg\,km^{-1}}$)**										
Gasoline PC	2	83.71	93.11	106.16	59.78	66.50	75.82	64.78	72.06	82.15
	3	70.25	73.16	75.90	54.73	57.00	59.14	61.18	63.72	66.11
	4	67.36	70.36	73.17	47.60	49.72	51.70	51.37	53.66	55.80
	5	54.48	56.66	59.57	36.35	37.80	39.75	38.35	39.88	41.93
	6	42.77	44.48	46.30	28.35	29.48	30.69	29.82	31.02	32.28
Diesel PC	6	0.08	0.94	2.03	0.07	0.77	1.66	0.07	0.88	1.88
Diesel LCV	6	1.34	3.88	7.52	0.87	2.52	4.88	0.91	2.63	5.09
